# 35 Hz shape memory alloy actuator with bending-twisting mode

**DOI:** 10.1038/srep21118

**Published:** 2016-02-19

**Authors:** Sung-Hyuk Song, Jang-Yeob Lee, Hugo Rodrigue, Ik-Seong Choi, Yeon June Kang, Sung-Hoon Ahn

**Affiliations:** 1 Department of Mechanical & Aerospace Engineering, Seoul National University, Seoul, 151-742, Korea; 2 Institute of Advanced Machines and Design, Seoul National University, Seoul, 151-742, Korea

## Abstract

Shape Memory Alloy (SMA) materials are widely used as an actuating source for bending actuators due to their high power density. However, due to the slow actuation speed of SMAs, there are limitations in their range of possible applications. This paper proposes a smart soft composite (SSC) actuator capable of fast bending actuation with large deformations. To increase the actuation speed of SMA actuator, multiple thin SMA wires are used to increase the heat dissipation for faster cooling. The actuation characteristics of the actuator at different frequencies are measured with different actuator lengths and results show that resonance can be used to realize large deformations up to 35 Hz. The actuation characteristics of the actuator can be modified by changing the design of the layered reinforcement structure embedded in the actuator, thus the natural frequency and length of an actuator can be optimized for a specific actuation speed. A model is used to compare with the experimental results of actuators with different layered reinforcement structure designs. Also, a bend-twist coupled motion using an anisotropic layered reinforcement structure at a speed of 10 Hz is also realized. By increasing their range of actuation characteristics, the proposed actuator extends the range of application of SMA bending actuators.

The selection of an actuator for a specific application is generally made by comparing the implementation requirements and the performance of different actuators. The performance of the actuator includes the mode of deformation, the scale of the deformation, its output force, and its actuation frequency. In the case of a bending actuator, it is generally mainly characterized by its dimensions, bending deformation and actuation frequency. Although mechanical actuators have good performance, their implementation requirements and the use of multiple joints to produce smooth biomimetic motions disqualify them from a number of applications. On the other hand, bending actuators making use of smart materials suffer from a trade-off between their actuation frequency and their bending performance with faster actuators having increasingly smaller bending performance.

Piezoelectric materials rely on a coupling between the electric field and mechanical stress to produce deformations and bending actuators can be formed by bonding a piezoelectric plate together with a metal-ceramic layer forming a unimorph or two piezoelectric plates forming a bimorph^1^,^2^. This type of actuator have the highest actuating frequency of all smart material-based actuators, but they are limited by their small deformation as shown in Fig. 1 3,4,5,6,7. This is because the ceramic materials used in this type of actuator are very brittle and susceptible to fracture^8^,^9^ such that large strains in the structure can degrade their actuating performance. Although there is no information about their actuation speed, piezoelectric actuators showed up to deflections of 1.48 mm^10^,^11^.

Ionic polymer-metal composite (IPMC) actuators, which is a type of ionic electroactive polymers (EAP) actuator, rely on an ionic polymer whose surface is plated with a conductor where ions migrate towards one surface with an imposed voltage^12^,^13^. This ion migration causes bending of the polymer in one direction, and actuators capable of large bending deformations in the range of 26 mm maximum tip deformation have been reported as in Fig. 1 14,15,16,17,18,19,20,21,22,23,24,25,26,27,28. Brunetto *et al.* found that the actuator’s deformation is increased when the actuating speed is matched to the natural frequency of the structure, resulting in an actuator capable of bending deflections of 7.2 mm at 9.5 Hz^18^. Shahinpoor *et al.* performed experiments for frequencies ranging from 0.1 to 35 Hz and found that there was an increase in maximum tip deformation around both the first and the second natural frequencies^23^. Although IPMC actuators have been reported to be capable of large deformations^29^, most of the IPMC actuators exhibit relatively small maximum tip deformation at higher frequencies due the need to use a thinner matrix to reduce the traveling distance of the ions within the matrix, but this also reduces the maximum deformation of the actuator since the actuation force is proportional to the thickness of the actuator^23^,^30^,^31^.

Electronic EAPs are another category of EAPs that can be categorized according to their actuation mechanism as either electrostrictive (ferroelectric polymers) or electrostatic (dielectric elastomers)^32^.

In ferroelectric polymers, the strain is generated by reversible alignment of polar groups due to the applied electric fields, and Poly(vinylidene fluoride) (PVDF) based polymers is the most widely used due to large bending deformations^33^,^34^. Lee *et al.*^35^, Tzou *et al.*^36^ developed bending actuators using PVDF-based polymers. In the case of dielectric elastomers, the electrostatic attraction between conductive layers generate a compression in thickness and stretching in the area of the polymer film^33^. To generate a bending motion, multiple flat dielectric films are stacked and bending deformations up to 56 mm maximum have been reported^37^. Multiple materials have been used to make these actuator such as Maleki *et al.* using PDMS^38^ and Mutlu *et al.* using stretched dielectric film^39^. However, none of the literature surveyed by the authors report both the actuation frequency as well as the bending deformation for either of these actuators.

Shape memory alloy (SMA) elements with an applied pre-strain undergo a strain recovery in the range of 4–8% when undergoing heating by transforming from a martensite phase at low temperatures to an austenite phase at high temperature, and backwards during cooling. SMA has such advantages compared to other types of smart materials as a high power density, but it has also been often described as having a slow actuation speed, which is seen as a major hindrance for its adaptation in a wider range of applications^40^. The main limiting factor for its slow actuating speed is the heating and cooling speed of the SMA^41^, so various techniques have been investigated for improving the cooling speed of the SMA itself such as forced air convection^42^, the use of a heat sink^40^, cooling using a water channel^43^ and a heat pump^44^. Other types of systems have also been proposed to increase their actuation speed such as optimization of the applied current^45^, improved controller design^46^ and segmented SMA control^47^.

In order to produce a bending motion using SMA, SMA wires are embedded or installed at an eccentric position with regards to an elastic beam structure which converts the linear contraction of the SMA wire into a large bending deformation of the structure. The actuation speed of this type structure and its actuating performance is shown in Fig. 1 48,49,50,51,52,53,54,55,56. These SMA based bending actuators are advantageous since they have relatively larger maximum tip deformations than other types of bending actuator, but they are limited by their slow actuation speed (~0.33 Hz) in comparison to both PZT and IPMC actuators. This is because most of the studies done on SMA actuators have focused on the properties of the SMA itself, and were rarely focused on realizing the fast actuation of a bending actuator. Similar principles have been used to produce diverse types of motions such as bending, twisting, and coupled bending-twisting motions^57^,^58^,^59^.

This paper introduces a SMA-based bending actuator that is capable of both fast and large deformations can be realized in either the pure-bending or coupled bending-twisting modes of actuation. To do so, smart soft composite (SSC) actuator consisting of a soft matrix, multiple embedded SMA wires and an anisotropic layered reinforcement structure were fabricated.

Figure 1 shows the previous limits in terms of maximum tip deformation versus actuating frequency of bending actuators using SMA and how the current work pushes the limits of SMA bending actuators up to 35 Hz such that the actuators presented in this work are those with the largest deformations amongst the smart materials-based bending actuators within their range of actuation speed in the surveyed literature. Results for the actuating length versus the actuation frequency as well as results related to the behavior of the actuator are shown, and the resonance characteristics of the actuator are measured. A layered reinforcement structure design strategy to target a specific actuation frequency is presented with a model to predict the length of the actuator required to obtain a resonant frequency corresponding to this actuation speed. Then, a layered reinforcement structure design strategy is presented that allows the actuator to produce large bend-twisting coupled motions at fast frequencies is presented. Also, the method of control deformation of the actuator is presented to control the amplitude of the motion and to produce asymmetric bending deformations. Finally, the performance of the actuator with a tip payload mass is presented.

## Actuator Design

**Figure 2:**
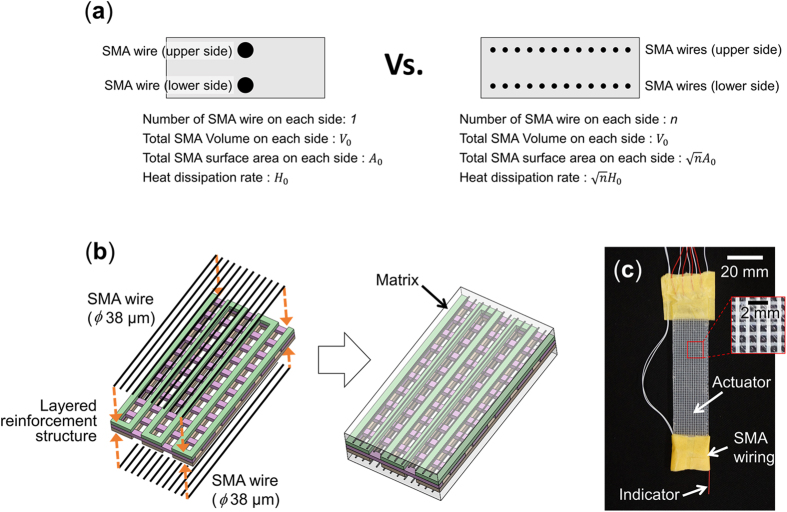
Design of the actuator. (**a**) Comparison of designs with one large SMA wire or multiple small SMA wires, (**b**) fabrication by 3D printing and casting process, (**c**) complete actuator.

The proposed actuator is a structure comprised of multiple SMA wires embedded in a polydimethylsiloxane (PDMS) matrix along the length of the actuator at positive and negative eccentricity with an acrylonitrile butadiene styrene (ABS) layered reinforcement structure embedded at the center. PDMS is thermally stable throughout the entire range of temperature during actuation of the SMA wires and presents good thermomechanical properties that make it suitable for use in soft actuators even though it, and many other polymers, is not the best choice of material to optimize cooling conditions. The SMA wires on one side of the matrix are actuated to bend the matrix towards that side, and the SMA wires on the other side of the matrix are actuated to bend the matrix towards the other side. Other types of bending actuators, such as IPMC or PZT actuators have made use of resonant amplification to realize large deformations at high speeds, but this type of strategy has not been tested on SMA-based bending actuators. The present work uses an SMA-based bending actuator where the actuation frequency corresponds to the resonant frequency of the actuator. However, since SMA wires actuate by phase transformation between the martensite and austenite states through changes in temperature, the actuator’s actuation speed is limited by the time required for changing temperature. Since SMA wires can be heated rapidly by Joule heating but cooling has to be done through convection or conduction, the cooling time is the main limiting factor. In order to increase the cooling speed of SMA wire to make use of resonant amplification of the actuator, the actuator is designed using multiple SMA wires with a small diameter rather than a single thick SMA wire (Fig. 2a) and its fabrication process is shown in Fig. 2b and Supplementary Fig. S1. This is due to the total force generated by SMA wires being proportional to the cross-sectional area of the wires but cooling rate being proportional to the surface area of the wires. Therefore, using multiple smaller wires with the same total cross-sectional area results in the same actuation force with an increased rate of cooling.

Furthermore, this actuator design facilitates the use of multiple small diameter SMA wires versus external actuation since all SMA wires have to be aligned precisely to facilitate the transfer of the contracting force to the structure and also, since SMA wires break easily at small diameters, the SMA wires within the matrix are protected from external forces that could damage them or lower their durability. Their alignment can also be set at a fixed distance between each SMA wires to optimize the cooling rate.

**Figure 3:**
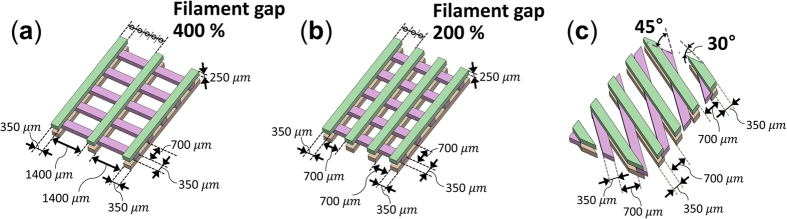
Design of the layered reinforcement structure. (**a**) Orthogonal layered reinforcement structure with large filament gap, (**b**) orthogonal layered reinforcement structure with small filament gap and (**c**) anisotropic layered reinforcement structure.

In order to be able to vary the actuating characteristics of the actuator, a layered reinforcement structure is embedded in the matrix whose configuration can be changed without making any other changes in the configuration of the actuator. The layered reinforcement structure is fabricated using a 3D printer for ease of design and manufacturing. Two layered reinforcement structure design strategies are presented in this work: the first allows changing of the natural frequency of the actuator, and the second allows the actuator to realize a bend-twist motion. To change the natural frequency of the actuator, the spacing of the elements in the layered reinforcement structure can be modified, two configurations are tested in this work where one has a large filament gap between layered reinforcement structure elements and the other a small filament gap, as shown in Fig. 3a,b. These two configurations have the same ply angle combination of [0/90/0] and the distance between each filament is only changed in the first and third layers to change only the longitudinal stiffness of the actuator. To realize a bend-twist coupled motion, anisotropic properties are added to the layered reinforcement structure by varying the angle of the layers of the layered reinforcement structure. To realize a symmetric bend-twist motion, where the motion is the same whether the upper or lower set of SMA wires are actuated, a symmetric angle ply combination such as [*θ*_1_/*θ*_2_/*θ*_1_] is used. In this work, an actuator with *θ*_1_ and *θ*_2_ equal to 30° and 45°, respectively, is built and tested, as shown in Fig. 3c.

## Result and Discussion

### Actuating length and actuating frequency

**Figure 4:**
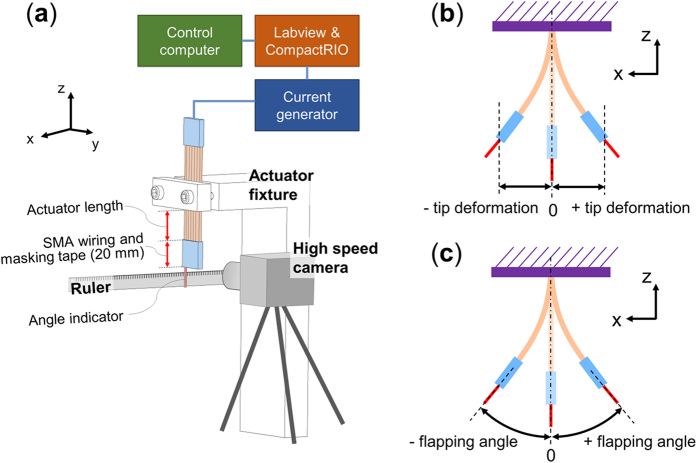
Setup and methods for observing the performance of the actuator. (**a**) Experimental setup, (**b**) Measurement of the tip deformation and (**c**) flapping angle.

The actuator’s length and its deformation magnitude are closely related since the deformation of the actuator is larger at a fixed radius of curvature for a longer actuator length. However, for high speed actuation, this might not be the case due to interaction with its environment and due to the decreased natural frequency associated with a longer length of the actuator. Furthermore, since the temperature and the deformation are coupled rather than the voltage and the deformation, the cooling time might have an effect on the maximum actuation angle. To verify these relations, experiments are conducted to measure the relation between the actuator’s length and its actuation frequency as in Fig. 4.

**Figure 5:**
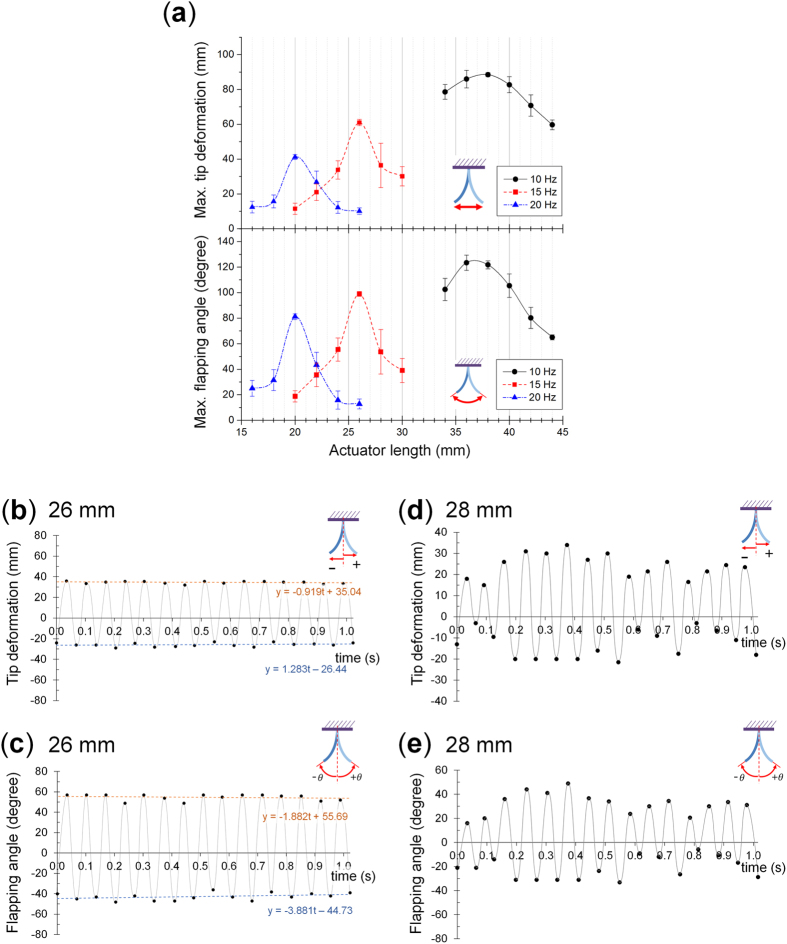
Actuating performance of actuator according to the actuator length. (**a**) Actuator maximum tip deformation and flapping angle for different actuation frequencies and actuation lengths. (**b**) Tip deformation and (**c**) flapping angle of the actuator with a length of 26 mm at 15 Hz. (**d**) Tip deformation and (**e**) flapping angle with a length of 28 mm at 15 Hz.

**Figure 6:**
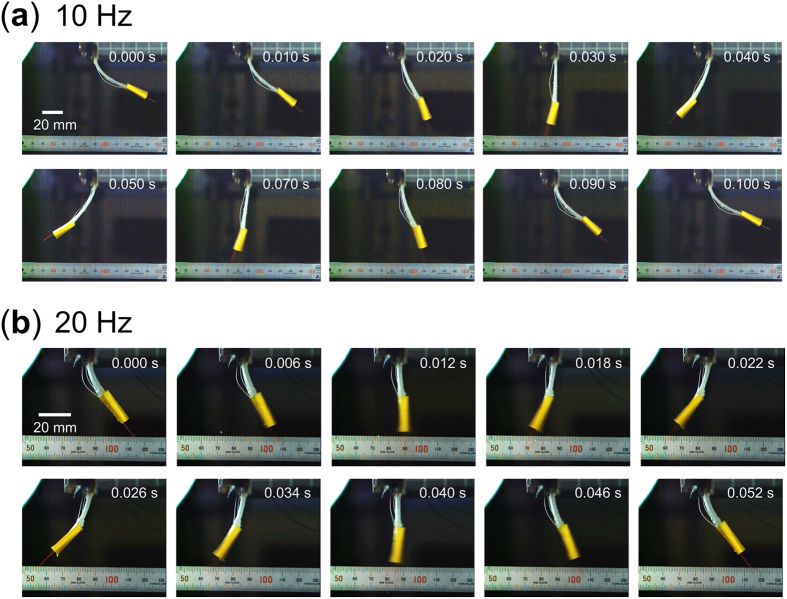
Deformation of the actuator with large filament gap in the layered reinforcement structure. Actuators actuation of a speed of (**a**) 10 Hz and (**b**) 20 Hz.

The length of the actuator where the embedded layered reinforcement structure has a large filament gap was varied in increments of 2 mm for actuation frequencies of 10, 15 and 20 Hz, and the maximum tip deformation and maximum flapping angle at each lengths were measured (Fig. 5a) and the shape of the actuator for actuation speeds of 10 and 20 Hz are shown in Fig. 6. For each frequency, there are noticeable peaks in both the maximum tip deformation and flapping angle at specific actuator lengths. This shows that different actuation frequencies require different actuator lengths to obtain the largest deformation and that since the deformation is increased significantly at specific lengths, it can be assumed that the resonance is the main influence behind this behavior.

It can also be seen that as the actuator length gets closer to the actuator length with a resonant frequency corresponding to the actuation speed, which is where the largest deformation can be observed, the error in data increases significantly and then diminishes nearly entirely at this actuator length. These characteristics correspond to those observed in the beating phenomenon and are another evidence that the actuating characteristics are influenced significantly by the resonance. This is shown more clearly in Fig. 5b–d where the maximum tip deformation and flapping angles for multiple cycles at 15 Hz are shown for lengths of 26 mm, which shows the highest deformation, and 28 mm which shows the largest error. In the actuator with actuator with a length of 26 mm, it can be seen that the actuator has a steady actuating performance. However, although it has a longer length, the actuator with a length of 28 mm shows a smaller average and maximum deformation, and also displayed large fluctuations corresponding to the beating phenomenon.

Since the resonance appears to be one of the main factors to determine the performance of the actuator, its motion can be described as a forced vibration system. Furthermore, since the deformation of the actuator shows a steady bending performance instead of increasing continuously to larger values, it can be described as a damped system. To check this assumption, the behavior of actuator in the transient region at 20 Hz is measured from the start of the actuation as shown in Supplementary Fig. S3. The behavior shown by the actuator is similar to that of a 1-DOF damped harmonic oscillator where the actuator starts with a small deformation and increases until the amplitude is limited by the damping force of the actuator. This actuation behavior is different from other SMA-based bending actuators where current is applied until the maximum actuation deformation at each cycle.

### Modal Analysis of Actuator

For verification of the previously obtained results, the natural frequency of the actuator can also be measured directly from experimental modal analysis using an electromagnetic shaker. Experiments were conducted for actuators with lengths of 20, 26 and 38 mm where the layered reinforcement structure has a large filament gap, which corresponds to the actuator lengths with the highest maximum deformations for actuation speeds of 10, 15 and 20 Hz as found previously. The tip displacements for the three different lengths of the actuators are measured by varying the input frequencies. Modal analysis is done only for the first mode since the intended actuating motion of the actuator is that corresponding to this mode. Supplementary Fig. S4 shows that natural frequencies of the actuators with lengths of 20, 26 and 38 mm are resulted in 8.5, 12 and 16.5 Hz, respectively. This differs slightly from the resonant frequencies found through actuation of the actuator, which can be explained by the difference in phase of SMA wires. During actuation, the SMA wires are heated and cooled repeatedly, such that the SMA wires can either be fully or partially in the austenite state which has a higher stiffness than the stiffness of the martensite state. However, the SMA wires should not be activated during the modal analysis since actuating the SMA wires would results in movements of the actuator not related to vibration from the exciter, such that the SMA has to be in the fully martensite state resulting in a slightly lower stiffness than during actuation. Although the resonant frequency of actuator during actuation is slightly higher than the natural frequency measured in the modal analysis, this experiment gives a good approximation of the natural frequency of the actuator during actuation.

### Layered Reinforcement Structure Design for Tailoring of Actuation Characteristics

One of the principal advantage of SSC actuators is that it is possible to change the actuation characteristics of the actuator by changing the layered reinforcement structure. Without making any changes to the configuration of the SMA wires, the material of the matrix or in the manufacturing method, it is possible to change the overall properties of the actuator by changing only the ply combination of layered reinforcement structure and therefore change its actuation characteristics. This section will show how the design of the layered reinforcement structure can be modified to change the actuation characteristics of the actuator.

#### Actuator Natural Frequency Design

Previous experiments showed that, with a fixed layered reinforcement structure design, the actuating length of the actuator can influence to the deformation magnitude and the actuator shows its highest deformation when it matches to the natural frequency of the actuator with that of the intended actuating speed. In this experiment, the stiffness of actuator is changed by changing the design of the layered reinforcement structure of the actuator and comparing the actuating characteristics of different layered reinforcement structure designs. Two different layered reinforcement structures configurations are tested in this work, which are those shown previously in Fig. 3a,b where one has a large filament gap of 400% the width of the layered reinforcement structure filament between filaments in the first and third layers and the other a small filament gap of 200%. The actuator with a large filament gap thus contains less ABS and has a lower stiffness than the actuator with a small filament gap, which should be helpful in realizing large deformation at small sizes for a specific actuation frequency range. However, as the actuation frequency is increased, the actuator length required to match the natural frequency becomes shorter, which reduces the active length of the SMA wire in the actuator available to produce deformations. Due to this conflict, it can be assumed that there is a specific frequency range for the stiffness of each layered reinforcement structure to realize large deformation for small size actuators.

**Figure 7:**
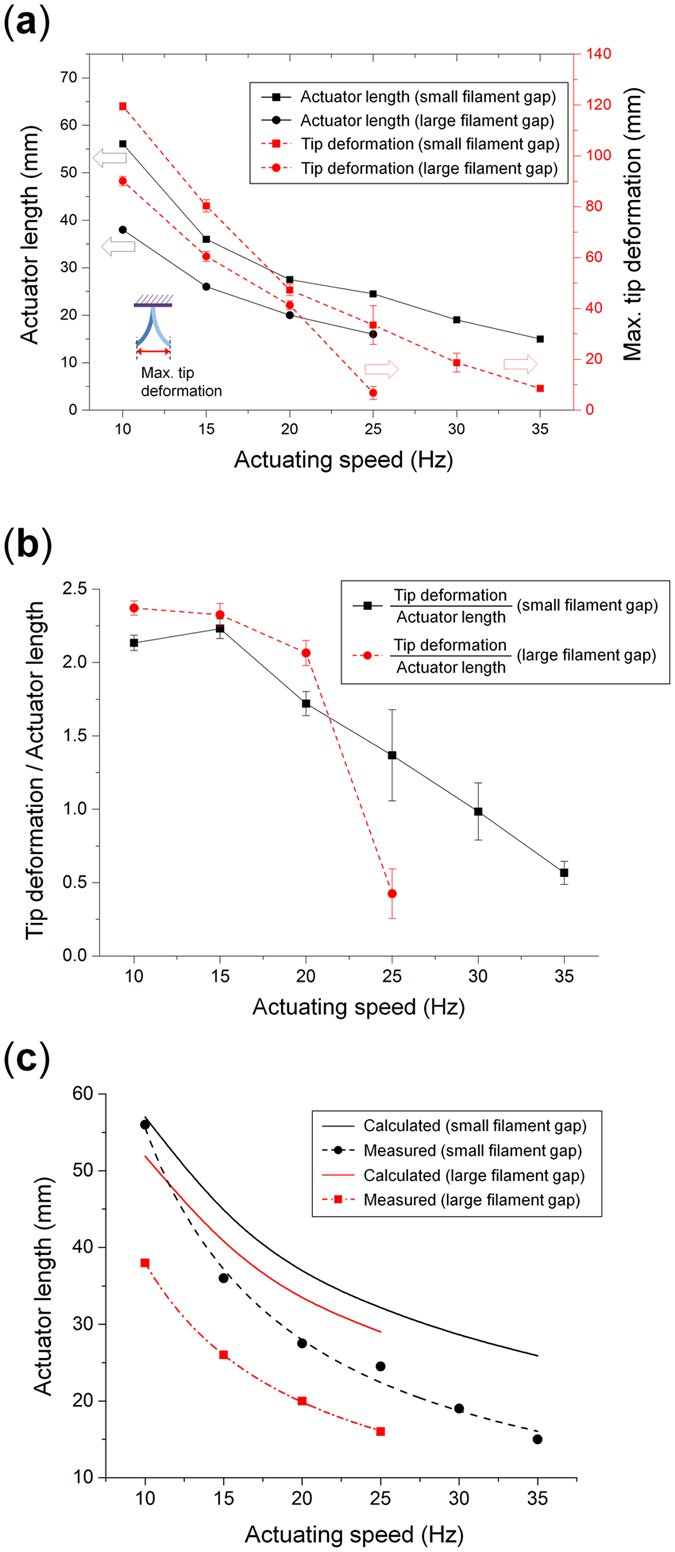
Actuating performance according to the layered reinforcement structure deign. (**a**) Actuating speed and maximum tip deformation for both layered reinforcement structure designs. (**b**) Actuation speed and maximum tip deformation per actuator length for both layered reinforcement structure designs. (**c**) Comparison of calculated and measured values for both layered reinforcement structure designs.

Therefore, to compare the actuating characteristics of the two layered reinforcement structure designs, the length of each actuator where the resonant frequency of the actuator corresponds to actuating speeds of 10, 15, 20, 25, 30 and 35 Hz are obtained as previously done. The results of this experiment are shown in Fig. 7a. The maximum tested actuation frequency of the actuator with a large filament gap is 25 Hz because the tip deformation became very small at actuation frequencies above 25 Hz. As predicted, the actuator with a small filament gap shows a longer length of the actuator to match the resonant frequency of the actuator with the actuation speed across all tested frequencies, which results in larger maximum tip deformations.

However, in certain applications there is a need for maximum performance over a specific length, in which case the maximum tip deformation per length becomes a critical parameter. The measured data is shown in Fig. 7b in terms of the maximum tip deformation per actuator length. These results show that the actuator with a large filament gap has a higher deformation per length at frequencies at or below 20 Hz while above 20 Hz the actuator with a small filament gap actuator performs better. These results show that different layered reinforcement structure designs perform better for different parameters, and that there is no single best design. Therefore, the selection of the filament gap of the layered reinforcement structure will depend on multiple parameters depending on the target application and its requirements.

Next, modeling of the actuation frequency based on filament gap is presented. Predicting the actuator’s required actuator length for a specific layered reinforcement structure design is important in order to match the target actuator length and the required deformation for the intended application.

As shown in Fig. 3, the natural frequency of the actuator can be controlled through the design of the configuration of the filament gap, so there is a need to calculate the required actuator length at a specific actuation frequency. In this paper, classical laminate theory and the beam vibration model are used to predict the resonant frequency for different actuators lengths and for each filament gap designs.

The actuator has a clamping section for the wiring which is attached at the end of the actuator with a mass *m* of 1.3 g. The mass of the clamping section is concentrated at the tip of the SMA wires, so it can be simplified as a point mass. Therefore, the actuator can be described as a cantilever beam with an end mass *m*, an actuator length *ℓ*, actuator mass density *ρ*, actuator width *b*, actuator thickness *t*, area moment of inertia of actuator *I* and a natural frequency *f* with the following relations^60^:


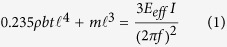


*E*_*eff*_ is effective bending modulus of actuator, and its calculation is shown in the Supporting Information. The results are then compared with the previously obtained results as shown in Fig. 7c. The measured value is the actuator length which shows the highest maximum tip deformation at a given actuation frequency and applying the formula for a range of values allows to obtain the relationship between the actuator length and the natural frequency of the actuator. Although the model will need to be improved in order to predict the length and natural frequency of the actuator, the trends of the numerical model and of the experimental results are in agreement.

#### Bend-Twist Coupled Mode Design

**Figure 8:**
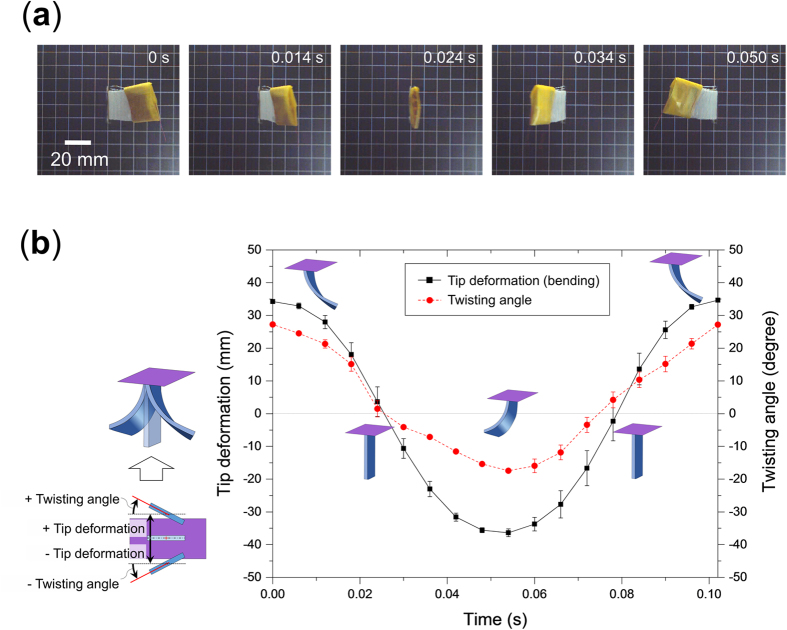
Bending-twisting coupled mode of actuation. (**a**) Photographs showing the bending-twisting coupled behavior of an actuator with a [30/45/30] layered reinforcement structure at an actuation speed of 10 Hz. (**b**) Tip deformation and twisting angle of the bending-twisting coupled mode actuator at an actuation speed of 10 Hz.

In addition to being able to adjust the natural frequencies through the filament gap design in the orthotropic ply configuration, the configuration of the layered reinforcement structure of the actuator can be modified to give it anisotropic properties, and thus produce a combined bending and twisting deformation. The layered reinforcement structures used in this work have three layers and their orientation were previously in the [0/90/0] orientation, which does not give any bend-twist coupling. In this experiment the layered reinforcement structure configuration was changed to [30/45/30], which gives the actuator bend-twist coupling resulting from its anisotropic properties. This actuator was tested at 10 Hz and an actuating length of 26 mm was determined to match the natural frequency of the actuator at this actuation frequency. Results for a complete cycle during 0.1 s showing the tip deformation and twisting angle of this actuator at this frequency is shown in Fig. 8. Results show that this actuator produces large bending deformations that are coupled with a large twisting deformation.

### Actuator Deformation Control

**Figure 9:**
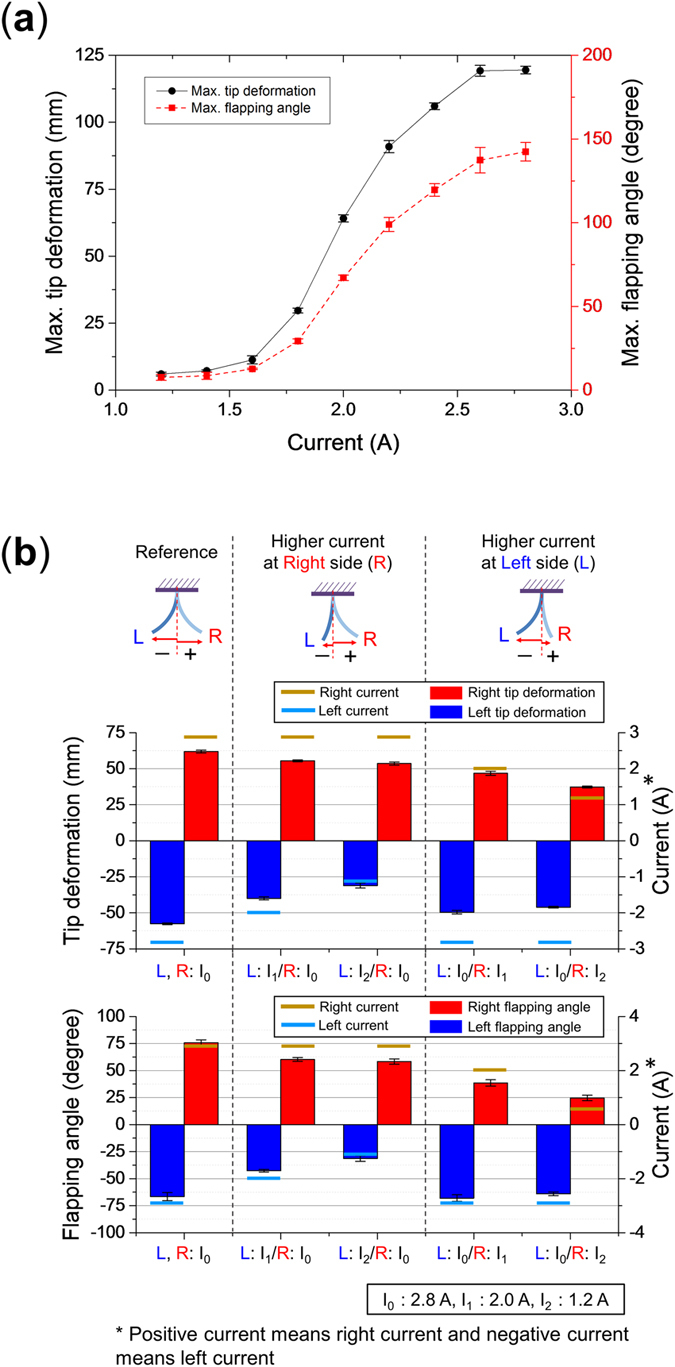
Control of the amplitude of deformation. (**a**) Control of the amplitude by changing the applied current and (**b**) asymmetric bending deformations using asymmetric applied current.

The deformation of the actuator can be varied by changing the current applied to the SMA wire without any effect on the actuation stability and on the natural frequency of the actuator. To verify this an experiment was conducted for an actuator with a length 56 mm, a small filament gap and using a fixed frequency of 10 Hz while the range of bending deformations were measured for applied currents ranging from 1.2 to 2.8 A. As the applied current to the SMA wire increased, the maximum tip deformation and maximum flapping angle are increased as shown in Fig. 9 (a). The error remains constantly small for all applied currents, which shows that the deformation of the actuator can be controlled independently of the frequency of the actuator. This is in contrast with the length of the actuator where the actuation speed and natural frequency of the actuator need to be matched to obtain a stable actuation pattern.

The deformation can also be controlled such that the actuator bends more towards one direction than the other by applying a different current for each direction of actuation. This was tested using the configuration of the previous experiment for different applied currents of 1.2 A, 2.0 A, 3.8 A with different combinations of these applied currents of the left and right sides. The results of this experiment are shown in Fig. 9 (b). If the same magnitude of current I_0_ is applied to the both left and right side of SMA wires (L, R: I_0_), the actuator shows a symmetrical large tip deformation and flapping angle in both directions. However, when a smaller current of 2.0 A is applied to the left side and a current of 3.8 A is maintained to the right side (L: I_1_/R: I_0_), then right side shows a larger deformation than the left side. If the smaller current is further decreased to 1.2 A (L: I_2_/R: I_0_), then the difference in bending amplitude between the left and right deformations is further increased. It is to be mentioned that there is a reduction in bending amplitude even on the side where the applied current is maintained at the higher current, but that it is much smaller than on the other side such that this represents a viable strategy to produce an asymmetric deformation. Similar results were also obtained for inversed current input for the left side. This shows that the current can be used to control both the bending amplitude and to introduce an imbalance in bending amplitude between both sides of the actuator.

### Payload Effect on the Actuator Performance

**Figure 10:**
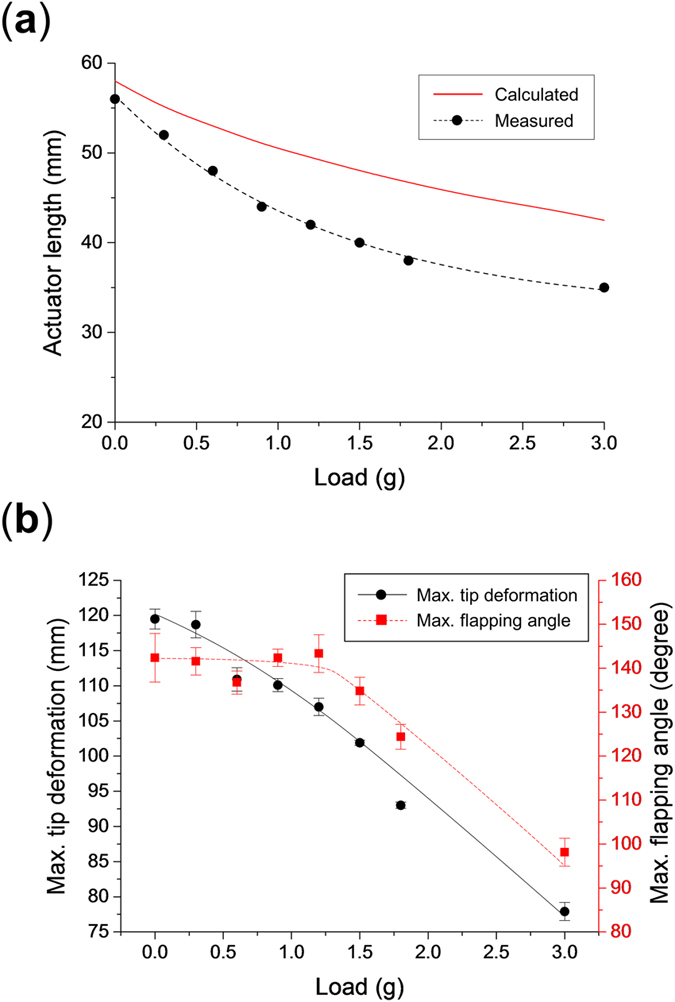
Effect of a payload tip mass on the performance of the actuator. (**a**) Effect on the actuator length required for a natural frequency of 10 Hz and (**b**) on the maximum tip deformation and flapping angle.

In order to see the effect of a payload on the actuator performance, the maximum tip deformation and maximum flapping angle were measured for different loads applied at the tip of the actuator. The weight was attached directly at the tip of the actuator and the actuator length that corresponds to a resonant frequency of 10 Hz was obtained experimentally, and the experiment was repeated for payload weights ranging 0.3 g to 3.0 g. As the payload is increased, the actuator length corresponding to a natural frequency of 10 Hz decreases as shown in Fig. 10(a). This is due to the natural frequency of the actuator changing due to the added mass, so the expected actuator length correspond to the resonance can be calculated using the modified equation (2) for a different payload mass *m*_*l*_ as follows.


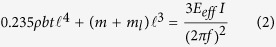


The results of this model for the tested actuator to obtain a natural frequency of 10 Hz with different payload masses is also shown Fig. 10(a). The results for the maximum tip deformation and flapping angle is shown in Fig. 10(b) where it can be seen that the tip deformation decreases steadily while the flapping angle stays constant until a payload mass of 1.2 g and then diminishes. These results show that the payload mass has an effect on the actuation properties, but that the design is able to perform well even with a non-significant payload mass and that it is possible to predict the effect of this added mass on the natural frequency of the actuator.

## Conclusion

A novel SSC actuator is presented in this work that combines multiple SMA wires with small cross-sections, a soft polymeric matrix and an ABS layered reinforcement structure where the actuation speed of the actuator corresponds to its natural frequency and the resonant amplification of the actuator increases the amplitude of actuation of the actuator such that large deformations will occur. The actuator requires a few actuation cycles to reach and then maintain its maximum tip deformation. This type of actuator was demonstrated to be capable of large deformations at actuation speeds up to 35 Hz, which is two order higher than the fastest SMA-based bending actuator found in the surveyed literature which had an actuation speed of 0.3 Hz. It is also the smart material-based soft actuator with the largest deformations in the 10–35 Hz range within the surveyed literature.

It was found that the actuating length of the actuator should be shortened for faster actuating speeds in order to increase the natural frequency of the actuator to match the actuating frequency. However, tailoring of the configuration of the layered reinforcement structure within the actuator allows to modify the actuation characteristics of the actuator and thus to target a specific actuator length, tip deformation and actuation frequency. Furthermore, the configuration of the layered reinforcement structure can also be changed to induce anisotropic properties to the actuator and thus produce a motion with a coupled bend-twist deformation. Although the actuation frequency of the actuator cannot be changed during use, the maximum tip deformation and maximum flapping angle could be controlled by changing the applied current. The performance of actuator for different loads were also measured and the expected actuator length corresponds to the resonance at given loaded condition could be calculated by proposed model. Also, from the small deviation of deformation, it could be verified that the hysteresis of SMA did not have a significant effect on the behavior of the actuator. However, the relatively high energy consumption (≤50 W) of the developed actuator could pose limitations for certain types of applications.

Future work will focus on the dynamic modeling of the actuator including the temperature of the SMA wires. By combining the model of the SMA with a modal analysis of structure, it should be possible to predict the actuation performance. Also, a real-time, precise and fast temperature measurement system for the actuator will be used to validate the results from the model. Other optimization avenues such as changing the material of the matrix and the use of SMA coatings will be investigated in order to further improve the cooling speed of the actuator and to reach higher actuation speeds. The developed actuator capable of fast bending actuation will also be applied to various fields where fast actuation speeds with large deformations is required such as flapping based flying robots or underwater swimming robots.

## Methods

### Actuator performance measurement

The actuator is fixed to a fixture which clamps it at a specific position along the length of the actuator, which allows to obtain a specific active length of the actuator. The actuator is then actuated using a current generator to generate the current patterns that is controlled using Labview and a CompactRIO real-time controller. The applied current magnitude is 2.8 A for all SMA wires with the square wave current pattern shown in Supplementary Fig. S2. The start and end time for the actuation of each set of SMA wires depends on the cycle time T of the intended actuation frequency. A ruler is fixed below the actuator which is used to measure the deformation in both bending directions, and a high speed camera (Ultima APX-RS, by Photron) set at 500 frames per second (FPS) is used to record the deformation of the actuator. The deformation magnitude is defined as the tip deformation in each bending direction, and the maximum tip deformation is defined as the sum of the tip deformations in both directions. The flapping angle is defined in each bending direction and the maximum flapping angle is defined as the sum of the flapping angles in both directions. The tip deformations and flapping angles are measured visually from the obtained videos using the high speed camera. The video used to measure the performance of the actuator is collected after 2 seconds of actuation to allow the actuator to reach its steady state performance.

### Experimental modal analysis

The natural frequency of the actuator is measured at different actuator lengths using an exciter. The actuator is fixed at a desired length by fixing it using the fixture to the tip of the stinger. The exciter used during this test is a 4854 Modal Exciter (Bruel & Kjaer Instruments, Inc.) using a Macro-tech 1202 (Crown, Inc.) power amplifier. The sinusoidal excitation signal at each given frequency is controlled using the Pulse Labshop 16.0 software (Bruel & Kjaer Instruments, Inc.) and generated using the LAN-XI Type 3160 Front end (Bruel & Kjaer Instruments, Inc.). To measure the response of actuator at each frequency input, the high speed camera (Ultima APX-RS, by Photron) is used to measure the tip displacement of the actuator with respect to the actuator’s fixture.

## Additional Information

**How to cite this article**: Song, S.-H. *et al.* 35 Hz shape memory alloy actuator with bending-twisting mode. *Sci. Rep.* **6**, 21118; doi: 10.1038/srep21118 (2016).

## Supplementary Material

Supplementary Information

## Figures and Tables

**Figure 1:**
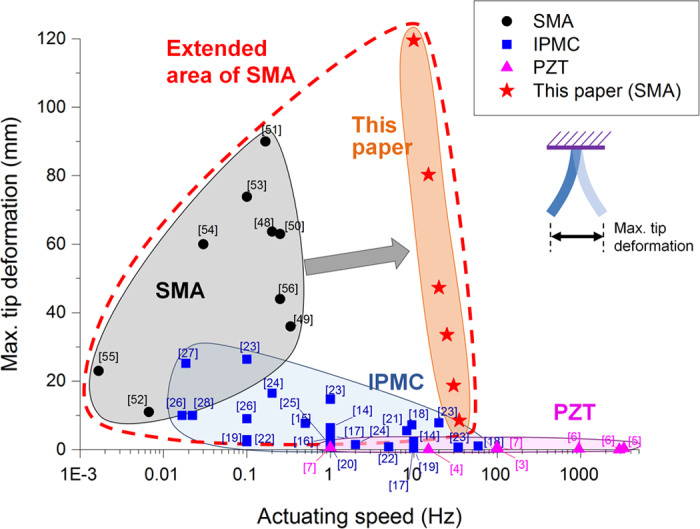
Comparison of the actuation speed and maximum tip deformation of bending actuators. The figure shows that the actuators in this paper extend the range of performance of this type of actuator such that they have the largest maximum tip deformation for larger actuation speeds than previously realized.
