# Supplementary material for: 35 Hz shape memory alloy actuator with bending-twisting mode
Source: Sci Rep. 2016 Feb 19;6:21118. doi: 10.1038/srep21118 (PMC4759690; doi:10.1038/srep21118)
Supplement: Supplementary Information [file srep21118-s1.pdf]

Supplementary Information

35 Hz shape memory alloy actuator with bending-twisting mode

Sung-Hyuk Song[1]**, Jang-Yeob Lee**[1]**, Hugo Rodrigue**[1], **Ik-Seong Choi**[1]**, Yeon June Kang**[1]**, and Sung-Hoon Ahn**[1,2,*]

[1]Department of Mechanical & Aerospace Engineering, Seoul National University, Seoul, 151-742, Korea

[2]Institute of Advanced Machines and Design, Seoul National University, Seoul, 151-742, Korea

*Correspondence and requests for materials should be addressed to S.H. Ahn (email: ahnsh@snu.ac.kr)

Supplementary Notes

Supplementary Note S1. Effective Bending Modulus of Actuator

The material properties of the SMA used in this paper shown in Supplementary Table S1[1]. The properties from the PDMS-layered reinforcement structure for the large filament gap and the small filament gap layered reinforcement structure designs along with the PDMS properties are shown in Supplementary Table S2. The Young's modulus (E^{PDMS}) and Poisson ratio (ν^{PDMS}) of PDMS are obtained from other literature[2,3] while the Young's modulus and Poisson's ratio of the different PDMS- layered reinforcement structure composite layers are obtained from a uniaxial tension test using a tensile testing machine (5948 Microtester, Instron, US). For this experiment, the specimens with longitudinal $[0°]_8$ and transverse $[90°]_8$ direction filaments with 400% and 200% filament gaps are prepared and tested according to the ASTM D3039/D3039M-14 standard. The state of the SMA wires alternates between the martensite and austenite states, it is therefore assumed that the SMA wires have an average stiffness between that of the fully martensite state and the fully austenite state for the calculations.

The lamina composition of the actuator is shown in Supplementary Fig. S5. Layers k = 1 and 7 consists of only PDMS and the layers k = 2 and 6 are PDMS-SMA wire composites. The layers k = 3, 4, 5 are PDMS-layered reinforcement structure composites so each layers in k = 3, 4, 5 correspond to the 1st, 2nd, 3rd layers of layered reinforcement structure. From classical laminate theory, the stiffness matrix of the actuator composed of the ABD parameter matrix can be represented as shown in Equation (S1) and calculated as shown in Equation (S2).

$$\begin{pmatrix} N_x \\ N_y \\ N_s \\ M_x \\ M_y \\ M_s \end{pmatrix} = \begin{pmatrix} A_{xx} & A_{xy} & A_{xs} & B_{xx} & B_{xy} & B_{xs} \\ A_{yx} & A_{yy} & A_{ys} & B_{yx} & B_{yy} & B_{ys} \\ A_{sx} & A_{sy} & A_{ss} & B_{sx} & B_{sy} & B_{ss} \\ B_{xx} & B_{xy} & B_{xs} & D_{xx} & D_{xy} & D_{xs} \\ B_{yx} & B_{yy} & B_{ys} & D_{yx} & D_{yy} & D_{ys} \\ B_{sx} & B_{sy} & B_{ss} & D_{sx} & D_{sy} & D_{ss} \end{pmatrix} \begin{pmatrix} \varepsilon_x^0 \\ \varepsilon_y^0 \\ \varepsilon_z^0 \\ \kappa_x \\ \kappa_y \\ \kappa_s \end{pmatrix} \tag{S1}$$

$$
\begin{aligned}
A_{ij} &= \sum_{k=1}^{n} Q_{ij}^{k} (h_k - h_{k-1}) \\
B_{ij} &= \frac{1}{2} \sum_{k=1}^{n} Q_{ij}^{k} (h_k^2 - h_{k-1}^2) \\
D_{ij} &= \frac{1}{3} \sum_{k=1}^{n} Q_{ij}^{k} (h_k^3 - h_{k-1}^3) \\
i, j &= x, y, s
\end{aligned}
\tag{S2}
$$

Both of the actuators with large filament gap and small filament gap layered reinforcement structures have a symmetric crossply laminate configuration such that the coupling stiffness B becomes 0, the equation can thus be simplified as follows[4]:

$$
\begin{pmatrix} N_x \\ N_y \\ N_s \end{pmatrix} = \begin{pmatrix} A_{xx} & A_{xy} & 0 \\ A_{yx} & A_{yy} & 0 \\ 0 & 0 & A_{ss} \end{pmatrix} \begin{pmatrix} \varepsilon_x^o \\ \varepsilon_y^o \\ \gamma_s^o \end{pmatrix}
\tag{S3}
$$

$$
\begin{pmatrix} M_x \\ M_y \\ M_s \end{pmatrix} = \begin{pmatrix} D_{xx} & D_{xy} & 0 \\ D_{yx} & D_{yy} & 0 \\ 0 & 0 & D_{ss} \end{pmatrix} \begin{pmatrix} \kappa_x \\ \kappa_y \\ \kappa_s \end{pmatrix}
\tag{S4}
$$

Furthermore, the actuated only actuates by bending in the x direction such that:

$$
\kappa_y = \kappa_{xy} = 0 \tag{S5}
$$

Then, from Equation (S4) and (S5),

$$
M_x = D_{xx} \kappa_x \tag{S6}
$$

$$
M = M_x b = D_{xx} \kappa_x b \tag{S7}
$$

Where b is the width of actuator. In an isotropic beam under an applied bending moment M, the equation of motion is given by:

$$
M = EI \kappa_x \tag{S8}
$$

Therefore, from Equation (S7) and (S8), the effective bending modulus of actuator E_{eff} is derived as

$$
E_{eff} = \frac{12 D_{xx}}{h^3} \tag{S9}
$$

Where h is the thickness of the actuator.

To verifiy the validity of the model presented above, the bending stiffness of the actuator was measured using a three-point loading test according to the ASTM D 790-03 standard test method using a tensile testing machine (5948 MicroTester, Instron, US). The measured results show that the actuator with large filament gap has a bending stiffness of 0.292 mN·m^2 and the actuator with small filament gap a bending stiffness of 0.435 mN·m^2. These values are very close with those calulcated using the proposed model whose bending stiffness were 0.278 mN·m^2 for the actuator with a large filament gap and 0.418 mN·m^2 for the actuator with a small filament gap when the SMA is fully in martensite phase. Therefore, the model is able to predict the actual bending stiffness of the actuator including the layered reinforcement structure with a margin of error of 5% for the tested configurations. However, it is only possible to measure the bending stiffness when the SMA wires are not actuated due to this causing deflection of the beam. However, it is assumed that the stiffness of SMA wires have an average value between the martensite and austenite phases, thus the calculated bending stiffness of the actuator using equation (1) is 0.378 mN·m^2 for the actuator with large a filament gap and 0.518 mN·m^2 for the actuator with a small filament gap, which is slightly higher than the non-actuated actuator.

Supplementary Note S2. Fabrication Process of Actuator

The fabrication of this actuator relies on an ABS 3D printed mold fabricated using a fused deposition modeling (FDM) 3D printer (Dimension 768 SST, Stratasys) with guides on each end of the mold to position the SMA wires within the matrix (Supplementary Fig. S1). The guides allow for the positioning of the SMA wires both below and above it by applying tension to the SMA wires and then fixing them to an external jig. The layered reinforcement structure is positioned in between the two sets of SMA wires after positioning of the lower set of SMA wires, and then the upper SMA wires are positioned. The actuator has 24 embedded SMA wires (Flexinol, Dynally Inc.) with a diameter of 38 μm where 12 have a negative eccentricity being positioned below the guide and 12 have a positive eccentricity being positioned above the guide. Then, PDMS (Sylgard 184, Dow-Corning) mixed with a ratio of 10:1 monomer to hardener is degassed, poured into the mold and cured at 50 °C for 12 hours. After curing of the PDMS, the actuator is removed from the mold, the SMA wires on each side are clamped using a thin copper rod and electric wires are soldered to the clamped part. Then the actuator is covered by a wet tissue

(Science Wipers Medium 41117, Kimtech) to further increase the cooling coefficient. The dimensions of the actuator are kept constant throughout all actuators at 20 mm in width, 70 mm in length and 1.8 mm in thickness with a total weight of 3.15 g excluding the wire clamps and the electric wires.

The layered reinforcement structure is fabricated using the same FDM 3D printer (Dimension 768 SST, Stratasys) as the mold because it is easy to fabricate the layered reinforcement structure using this process. The filament dimension of the layered reinforcement structure is 350 μm in width by 250 μm in thickness, which is same as that deposited by the nozzle of the 3D printer.

Supplementary Figures

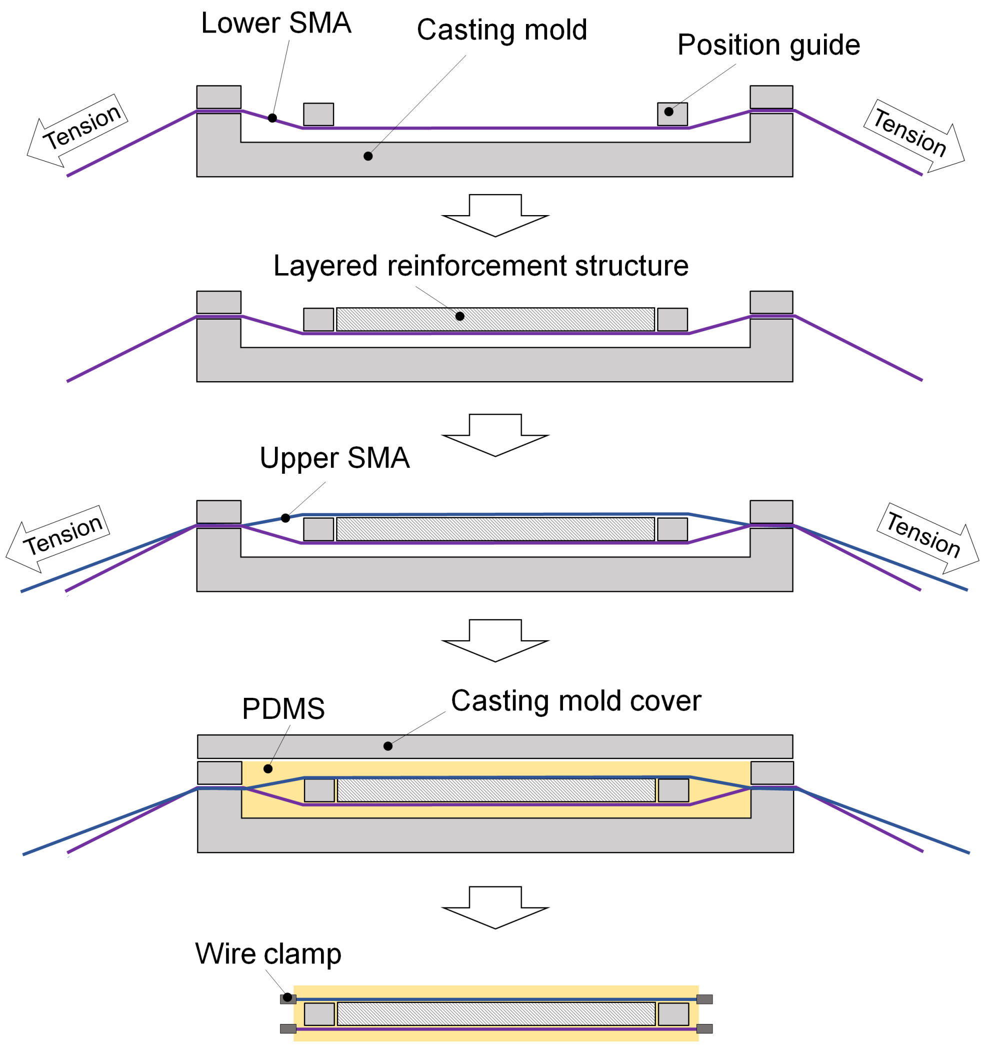

Supplementary Figure S1. Fabrication process of the actuator. A casting mold fabricated by 3D printer is used to fabricate the actuator that uses position guides to position the SMA wires. The layered reinforcement structure is inserted above the lower SMA wires before positioning the upper SMA wires.

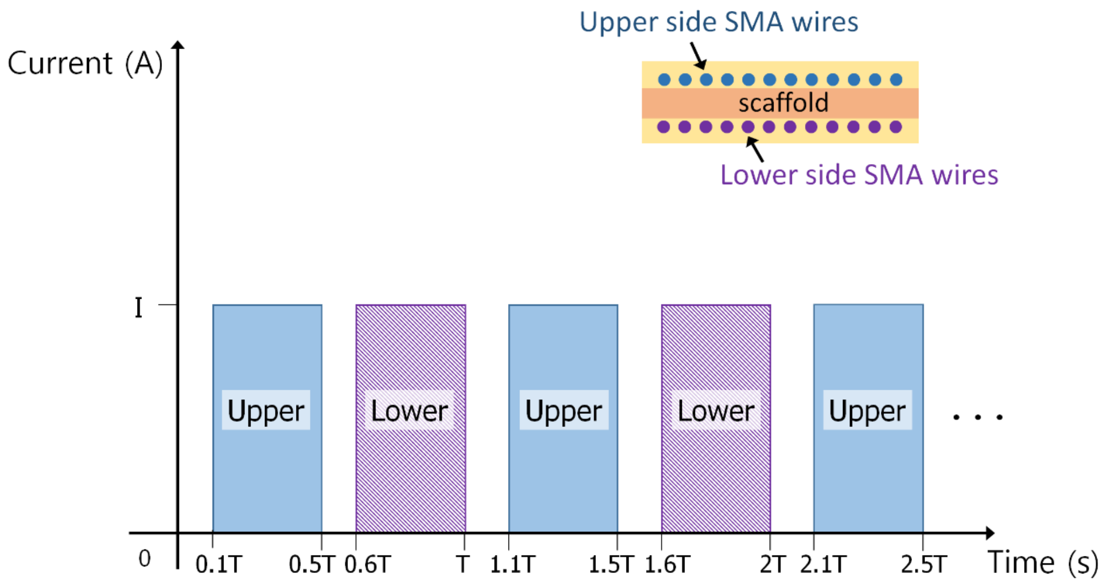

Supplementary Figure S2. Current input profile into the actuator. The blue sections correspond to current applied the upper side SMA wires and the purple sections to the lower SMA wires. The signal profile is a square wave form and the cycle time T is varied depending on the desired actuation speed.

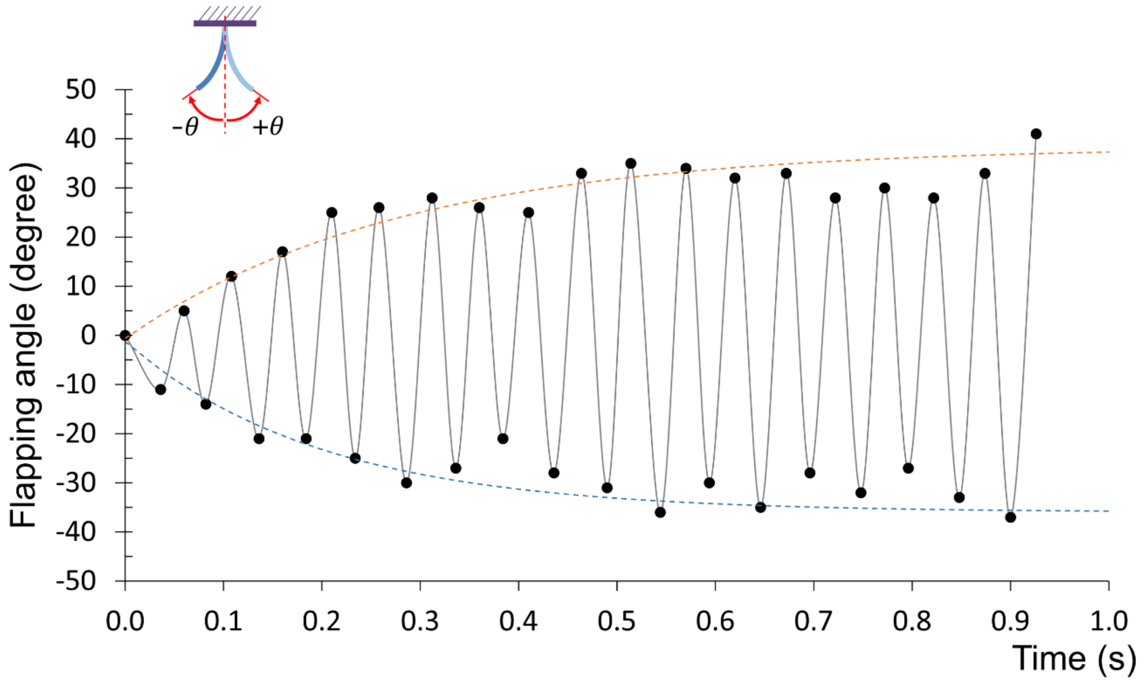

Supplementary Figure S3. Actuation characteristics of the actuator with a large filament gap layered reinforcement structure at the beginning of actuation at an actuation speed of 20 Hz. The actuating behavior is similar with the forced harmonic oscillator of a 1-DOF damped system and supports the hypothesis that the actuator behavior corresponds to a damped beam vibration under harmonic force.

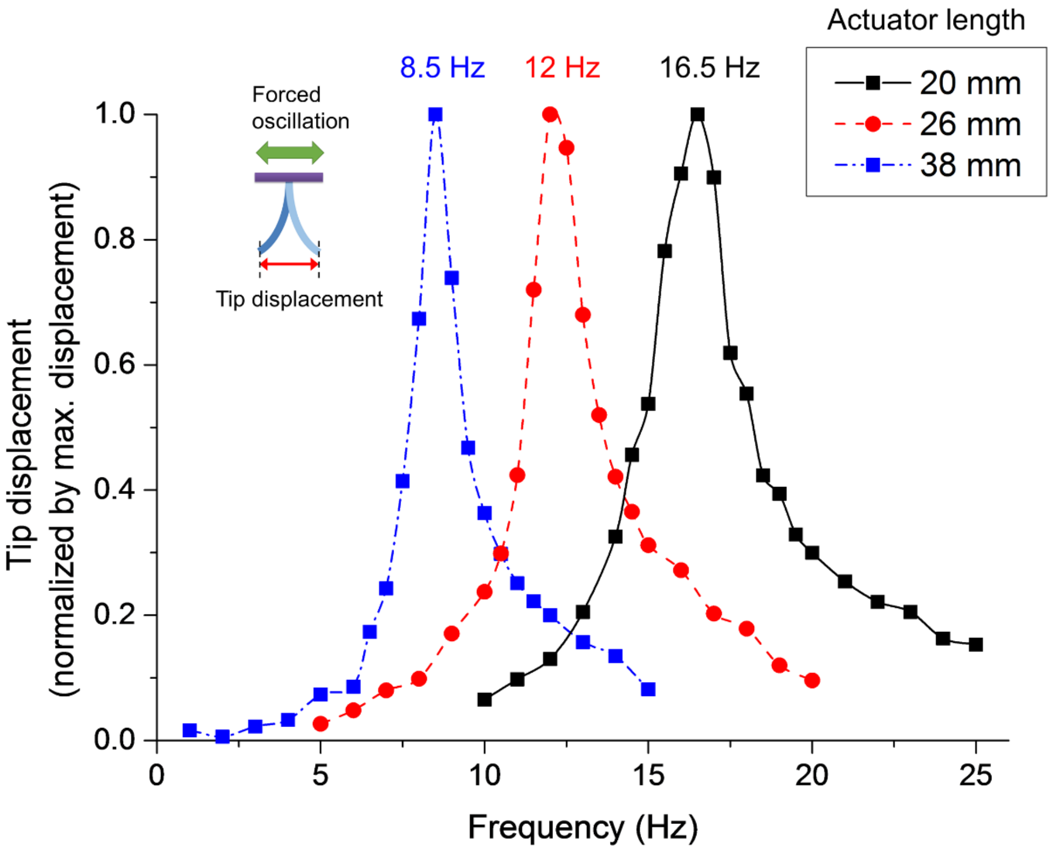

Supplementary Figure S4. Resonance characteristics for actuators with lengths of 20, 26 and 38 mm using an electromagnetic shaker.

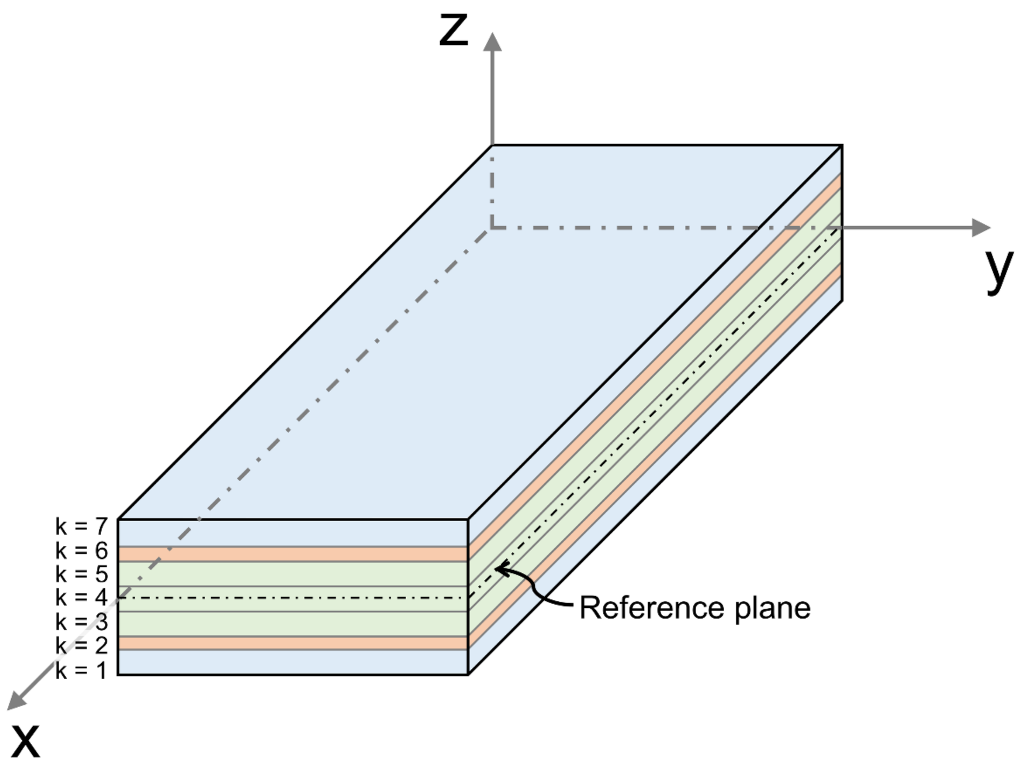

Supplementary Figure S5. The configuration of the lamina composition of the actuator for applying the model.

Supplementary Table S1. SMA material property

Parameter	Specification	Unit	Value
E^M	Young's modulus, Martensite phase	GPa	28
E^A	Young's modulus, Austenite phase	GPa	75
A^s	Austenite start temperature	K	351
A^f	Austenite finish temperature	K	341
G^M	Shear modulus, Martensite phase	GPa	10.56
G^A	Shear modulus, Austenite phase	GPa	28.20
v^s	Poisson ratio	—	0.33

Supplementary Table S2. Composite material property

Parameter	Specification	Unit	Value
E^{PDMS}	Young's modulus of PDMS	MPa	1.32
ν^{PDMS}	Poisson's ratio of PDMS	—	0.45
E_{1_s}	Young's modulus in longitudinal direction of PDMS – small filament gap layered reinforcement structure composite	MPa	420.8
E_{2_s}	Young's modulus in transverse direction of PDMS-small filament gap layered reinforcement structure composite	MPa	1.403
ν_{12_s}	Poisson's ratio of PDMS-small filament gap layered reinforcement structure composite	—	0.37
E_{1_l}	Young's modulus in longitudinal direction of PDMS – large filament gap layered reinforcement structure composite	MPa	214.0
E_{2_l}	Young's modulus in transverse direction of PDMS-large filament gap layered reinforcement structure composite	MPa	1.381
ν_{12_l}	Poisson's ratio of PDMS-large filament gap layered reinforcement structure composite	—	0.41

Supplementary References

1. Rodrigue, H., Wei, W., Bhandari, B. & Ahn, S.-H. Fabrication of wrist-like SMA-based actuator by double smart soft composite casting. *Smart Mater. Struct.* **24**, 125003 (2015).

2. Johnston, I., McCluskey, D., Tan, C. & Tracey, M. Mechanical characterization of bulk Sylgard 184 for microfluidics and microengineering. *J. Micromech. Microeng.* **24**, 035017 (2014).

3. Xia, Y. et al. Complex optical surfaces formed by replica molding against elastomeric masters. *Science* **273**, 347-349 (1996).

4. Daniel, I.M., Ishai, O., Daniel, I.M. & Daniel, I. Engineering mechanics of composite materials, Vol. 3. (Oxford university press New York, 1994).
